# Ultrasensitive Electrochemical Biosensors Based on Allosteric Transcription Factors (aTFs) for Pb^2+^ Detection

**DOI:** 10.3390/bios14090446

**Published:** 2024-09-18

**Authors:** Ningkang Yu, Chen Zhao, Xiaodan Kang, Cheng Zhang, Xi Zhang, Chenyu Li, Shang Wang, Bin Xue, Xiaobo Yang, Chao Li, Zhigang Qiu, Jingfeng Wang, Zhiqiang Shen

**Affiliations:** 1 College of Food Science and Technology, Shanghai Ocean University, Shanghai 201306, China; ynk0122@126.com (N.Y.); 13849918731@163.com (X.K.); 2 Military Medical Sciences Academy, Tianjin 300050, China; zhaochen212@126.com (C.Z.); 15062724512@163.com (C.Z.); zhangxi0820@126.com (X.Z.); nk_lcy710430@hotmail.com (C.L.); wsh847@163.com (S.W.); xue_bin04@163.com (B.X.); 18072712080@163.com (X.Y.); lc6628@163.com (C.L.); zhigangqiu99@gmail.com (Z.Q.)

**Keywords:** electrochemical, allosteric transcription factors (aTFs), PbrR, Pb^2+^

## Abstract

Exposure to Pb^2+^ in the environment, especially in water, poses a significant threat to human health and urgently necessitates the development of highly sensitive Pb^2+^ detection methods. In this study, we have integrated the high sensitivity of electrochemical techniques with allosteric transcription factors (aTFs) to develop an innovative electrochemical biosensing platform. This biosensors leverage the specific binding and dissociation of DNA to the aTFs (PbrR) on electrode surfaces to detect Pb^2+^. Under the optimal conditions, the platform has a broad linear detection range from 1 pM to 10 nM and an exceptionally low detection threshold of 1 pM, coupled with excellent selectivity for Pb^2+^. Notably, the biosensor demonstrates regenerative capabilities, enabling up to five effective Pb^2+^ measurements. After one week of storage at 4 °C, effective lead ion detection was still possible, demonstrating the biosensor’s excellent stability, this can effectively save the cost of detection. The biosensor also achieves a recovery rate of 93.3% to 106.6% in real water samples. The biosensor shows its potential as a robust tool for the ultrasensitive detection of Pb^2+^ in environmental monitoring. Moreover, this research provides new insights into the future applications of aTFs in electrochemical sensing.

## 1. Introduction

With the rapid development of industrialization and the intensification of human activities, heavy metal contamination in aquatic environments has become a pervasive and critical issue [1]. Among the various heavy metal ions, Pb^2+^ is one of the most harmful and prevalent pollutants in aquatic environments [2]. The non-biodegradable nature of Pb^2+^ allows it to persist in water systems, leading to bioaccumulation and biomagnification through the food chain, which can pose severe health risks to humans, particularly to children, by damaging critical organs such as the nervous system, reproductive system, and kidneys [3,4,5]. Given the significant health and environmental impacts of Pb^2+^, regulatory bodies worldwide have established stringent limits to control its concentration in drinking water. The World Health Organization (WHO) has set a maximum allowable level of 0.01 mg/L (48 nM) for Pb^2+^ in drinking water, while the United States Environmental Protection Agency (EPA) enforces a similar guideline to safeguard public health [6,7]. To mitigate the toxic effects of lead in water, the monitoring and detection of Pb^2+^ have become increasingly critical in recent years.

Currently, a variety of analytical techniques are employed for heavy metal detection, such as Inductively Coupled Plasma Mass Spectrometry (ICP-MS) [8], Inductively Coupled Plasma–Atomic Emission Spectroscopy (ICP-AES) [9], and Atomic Absorption Spectrometry (AAS) [10]. While these methods offer high sensitivity and selectivity, they require sophisticated and expensive equipment, time-consuming procedures, and skilled operators. Consequently, these techniques are not suitable for routine on-site rapid testing. Compared with traditional detection techniques, several innovative and rapid detection methods have been developed to date that can qualitatively or semi-quantitatively detect targets. Among these, electrochemical biosensors have garnered significant attention due to their high selectivity, sensitivity, simplicity, rapidity, and low cost [11,12,13,14]. These sensors have been widely applied in detecting various analytes, including nucleic acids [15], proteins [16], viruses [17], heavy metal ions [18], and small molecules targets [19]. Most electrochemical biosensors employ recognition elements such as aptamers and antibodies, which selectively react with the target analyte, generating an electrical signal correlated with the analyte concentration [20,21,22]. While antibody-based immunosensors are characterized by high sensitivity and specificity, they also suffer from high production costs and instability during storage and transport. Aptamer sensors can be tailored to selectively bind with a wide range of targets, but they require a complex screening process [23,24]. Therefore, it is essential to select recognition elements for electrochemical biosensors that are simple and easy to synthesize, possess strong binding affinity for the target, and exhibit high specificity.

Recently, allosteric transcription factors (aTFs) have been increasingly utilized in the field of biosensing as recognition elements for detecting chemical contaminants [25,26,27]. ATFs are naturally occurring regulatory proteins that contain a DNA-binding domain (DBD) and an effector-binding domain (EBD), modulate gene expression by undergoing conformational changes upon binding to specific ligands, enabling them to respond to chemical contaminants, and produce a detectable signal [28,29,30,31]. In contrast to aptamers, antibodies or other specific biomolecules, aTFs have advantages in terms of wide range of aptamer detection targets, high sensitivity and specificity of antibody recognition analytes [23,32,33,34]. Recent studies have utilized aTFs as biorecognition elements to develop electrochemical biosensors for the detection of small-molecule biomarkers and analytes [35]. PbrR is a well-known aTF belonging to the MerR family and has been identified as having superior Pb^2+^ recognition properties from *Cupriavidus metallidurans CH34* [27]. PbrR can bind to specifical binding site to form the PbrR-DNA complex and dissociate upon binding to Pb^2+^. The ability of PbrR to bind and dissociate from DNA establishes it as a novel and specific in vitro biosensing recognition element for Pb^2+^ [25,36].

In the present laboratory-based study, a cell-free paper-based biosensor using PbrR was designed for the detection of Pb^2+^, achieving a detection limit as low as 0.1 nM within 60 min [27]. In this work, we developed a PbrR-based electrochemical biosensor for the detection of Pb^2+^ in water, combining the high sensitivity of an electrochemical sensor with the specificity of aTFs. The presence of Pb^2+^ leads to the dissociation of the PbrR–DNA complex on the electrode, resulting in a change in the electrochemical signal that enables rapid on-site detection within a concentration range of 1 pM to 10 nM in under 10 min. The biosensor can be regenerated by re-incubation with PbrR, restoring its initial state and allowing it to be reused up to five times for subsequent detection cycles. This approach eliminates the need for complex sample preparation, significantly shortens testing time, and reduces testing costs through reuse. Moreover, as a universal detection platform, the electrochemical biosensors can be combined with other aTFs for the detection of specific targets, providing a new, efficient, and fast platform for the detection of heavy metal ions.

## 2. Materials and Methods

### 2.1. Materials and Reagents

Potassium hexacyanoferrate (II) trihydrate (K_4_[Fe(CN)_6_]·3H_2_O), potassium hexacyanoferrate (III) (K_3_[Fe(CN)_6_]), potassium chloride, magnesium chloride, alumina powder (1.0 µm, 0.3 µm, and 0.05 µm), anhydrous ethanol, hexamercaptohexanol (MCH), tris(2-carboxyethyl)phosphine hydrochloride (TCEP), TE buffer, and sodium dodecyl sulfate (SDS) were all obtained from analytical-grade reagents. All aqueous solutions were prepared using Millipore-Q water. All other reagents and solvents used were of analytical grade. All aqueous solutions were made with Millipore-Q water. 

The PbrR from *Cupriavidus metallidurans CH34* was expressed and purified by Biological Engineering Technology & Services Co., Ltd. (Nanjing, China), and the sequences are provided in Table S1. 

The SH-DNA was synthesized by Shanghai Sangon Biological Engineering Technology & Services Co., Ltd. (Shanghai, China), and the sequences are presented in Table S2.

### 2.2. Apparatus

All electrochemical measurements were conducted on an electrochemical workstation (Princeton P4000A, AMETEK, Berwyn, PA, USA) at room temperature. The experiments utilized a standard three-electrode system, consisting of a functionalized gold working electrode, an Ag/AgCl reference electrode with 3M KCl, and a platinum counter electrode. Details of the pre-treatment of gold electrodes can be seen in the Supplementary Materials (Text S1 and Figure S1).

Following the methodology of a previous study with minor modifications [35], the electrolyte contained 2 mM each of K_3_[Fe(CN)_6_]/K_4_[Fe(CN)_6_] in 10 mM Tris buffer (pH 7.6) with 100 mM KCl and 2.5 mM MgCl_2_. Cyclic voltammetry (CV) was performed over a potential range from −0.2 to 0.6 V at a scan rate of 50 mV/s. Electrochemical impedance spectroscopy (EIS) was conducted over a frequency range of 0.1 Hz to 100 kHz with an amplitude of 5 mV. Square-wave voltammetry (SWV) was also performed within the same potential range of −0.2 to 0.6 V. Due to its higher precision in quantitative analysis compared to other electrochemical methods, SWV was chosen for subsequent quantitative assays. 

### 2.3. Construction of Electrochemical Biosensors

Before immobilization on the gold electrode surface, the synthesized DNA, in the form of a dry powder, was dissolved to a concentration of 100 µM in TE buffer at pH 6.8. To prevent the formation of disulfide bonds from the sulfhydryl groups, the DNA was chemically reduced using TCEP. This process involved mixing the thiolated DNA with TE buffer containing 10 mM TCEP and incubating the mixture in the dark for 30 min to ensure complete reduction. The reduced SH-DNA single strand was mixed with the complementary ssDNA strand and heated in a water bath at 95 °C for 5 min, followed by a gradual cooling to room temperature to allow for the formation of a stable double-stranded DNA structure. The DNA duplex was then diluted to a final concentration of 10 µM with TE buffer and stored at 4 °C until further use.

The DNA self-assembled onto the electrode via Au–S bonding. The preserved double-stranded DNA with sulfhydryl groups was drop-coated onto the gold electrode with 10 µL, a process that was followed by air-drying at room temperature. The electrode was subsequently rinsed gently with TE buffer to remove any unbound DNA and dried under nitrogen. The resulting electrode was designated as Au/DNA. To prevent non-specific adsorption, 10 µL aliquot of 1 mM MCH was drop-coated on the electrode surface and incubated at room temperature for 1 h. After incubation, the electrode was rinsed with ultrapure water to remove any physically adsorbed MCH. The resulting electrode was designated as Au/DNA/MCH. Finally, PbrR was drop-coated onto the electrode and incubated at room temperature for 1 h to allow for binding. Excess PbrR was removed by rinsing the electrode with ultrapure water, resulting in the final electrode construct, designated as Au/DNA/MCH/PbrR. Each step of the electrode modification process was meticulously characterized using CV and EIS to confirm successful modification and functionalization of the electrode. In Text S3, atomic force microscopy (AFM) was used to investigate the roughness of the electrode modified by DNA and PbrR.

### 2.4. Optimization of Conditions for the Construction of Biosensors

The biosensor’s analytical performance was optimized by evaluating the immobilization of DNA at varying concentrations on the electrode, the concentrations of PbrR, and the incubation time of Pb^2+^.

DNA concentrations of 0, 1, 1.5, 2, 3, and 4 µM were drop-coated onto the electrode and subsequently blocked with MCH. The optimal DNA concentration was determined by analyzing the SWV signal responses, which varied with each DNA concentration. The concentration that elicited the most significant SWV signal change was identified as the optimal condition, thereby enhancing the sensor’s detection capability. 

To optimize the incubation concentration of PbrR with DNA, the DNA concentration was fixed at 1.5 µM, and different concentrations of PbrR (0, 1.5, 7.5, 15, 30, 45, 60 µM) were incubated on the electrode for 1 h. The optimal PbrR concentration was determined by observing the SWV signals generated at different PbrR concentrations.

For the rapid detection of Pb^2+^, a fixed concentration of Pb^2+^ was incubated on the electrode for 1, 5, 10, 15, 20, 25, and 30 min. The optimal detection time was determined by comparing the signal changes corresponding to each incubation period. 

### 2.5. Pb^2+^ Quantitative and Selectivity Evaluation

In order to evaluate the sensitivity of the biosensors for the detection of Pb^2+^, 10 µL of water sample with graded concentrations of Pb^2+^ (0, 1 pM, 10 pM, 100 pM, 1 nM, 10 nM) was applied dropwise to the prepared electrodes. To assess the selectivity of the proposed biosensor, 10 µL of the solutions containing other common metal ions, such as Cu^2+^, Ni^+^, As^3+^, Cd^2+^, and Hg^2+^, were added dropwise to the electrodes at a concentration of 0.1 nM. Additionally, a mixture of 1 pM Pb^2+^ with all the aforementioned heavy metals at 0.1 nM was also prepared, and 10 µL of this mixture was applied to the biosensor.

### 2.6. Regeneration and Stability of Biosensors

In order to further investigate the regeneration of the prepared electrochemical biosensors, the electrodes were restored to their initial state after each test using a regeneration solution. Specifically, 10 µL of 0.5% SDS was applied dropwise onto the electrode surface and incubated for 30 min to remove the aTFs specifically bound to the DNA. The electrode was then rinsed with ultrapure water. Afterward, 10 µL of 30 µM PbrR was drop-coated onto the electrode surface and incubated for 1 h before the target was added for regeneration testing. This regeneration process was repeated, and the signal changes were monitored.

To evaluate the stability of this biosensor, the PbrR-modified Au/DNA/MCH/PbrR electrode was stored at 4 °C and used for lead ion detection at weekly intervals, with signal changes observed over time.

### 2.7. Application of Biosensor for Pb^2+^ Detection in Actual Water Samples

The river water was collected from the Haihe River (Tianjin, China). Various concentrations of Pb^2+^ (5 nM, 7 nM, 10 nM) were added to river water for the recovery experiments. The actual water samples were filtered with a syringe filter, and the rest of the protocols were followed as outlined earlier. 

### 2.8. Statistics

All statistical analyses were performed using SPAW Statistics 18 software (SPSS Inc. Chicago, IL, USA). Heterogeneous data were analyzed using a non-parametric test such as the Friedman test. A *p*-value < 0.05 was considered statistically significant (* *p* < 0.05, ** *p* < 0.01, *** *p* < 0.001, and **** *p* < 0.0001).

## 3. Results and Discussion

### 3.1. Principle and Construction of the Electrochemical Biosensors

**Figure 1:**
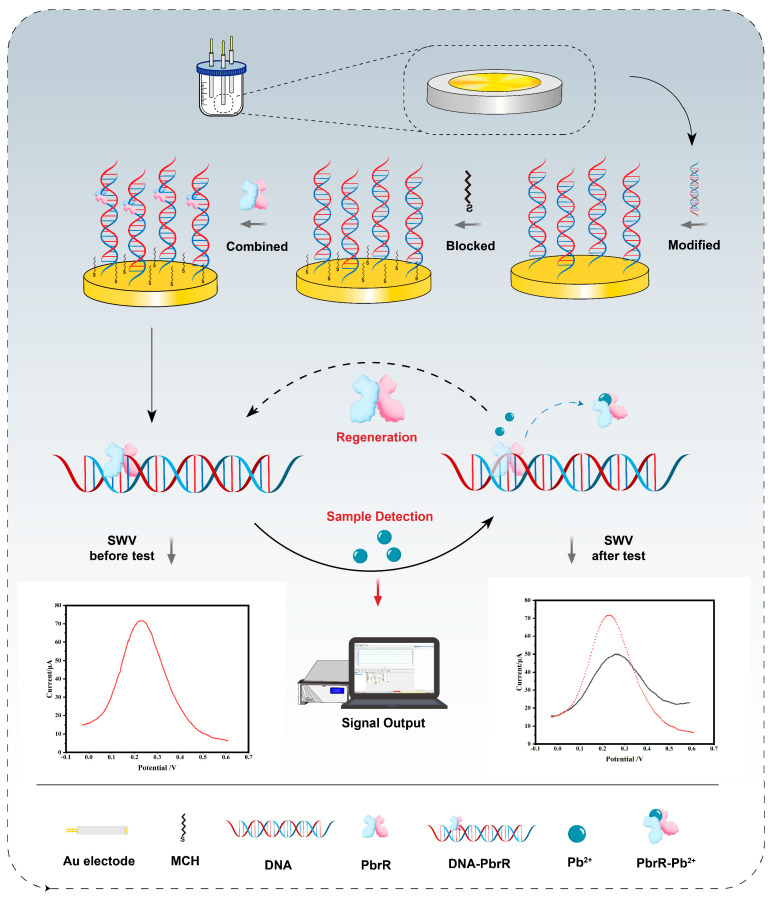
The principle of electrochemical biosensors based on aTFs.

This work focuses on the development of an electrochemical biosensor utilizing aTFs as recognition elements. Figure S2 demonstrates the ability of DNA to bind to PbrR using an electrophoretic mobility shift assay (EMSA). Figure 1 illustrates the underlying principles schematically. The biosensor construction involves several key steps as follows: First, DNA sequences containing PbrR binding sites were immobilized on the electrode surface via Au–S bonding. Second, the electrode’s non-specific adsorption sites were blocked with MCH. Third, PbrR was incubated on the electrode to bind the DNA, forming a DNA–PbrR complex.

During testing, the sample was added to the electrode surface. When Pb^2+^ came into contact with the DNA–PbrR complex on the electrode, it bound to PbrR and caused the dissociation of the complex from the DNA, triggering a change in the electrochemical signals. The concentration of Pb^2+^ was quantitatively detected by measuring the change in the SWV electrochemical signals. After each test, new PbrR was reapplied to the electrode surface for sensor regeneration, enabling the repeated detection of Pb^2+^.

**Figure 2:**
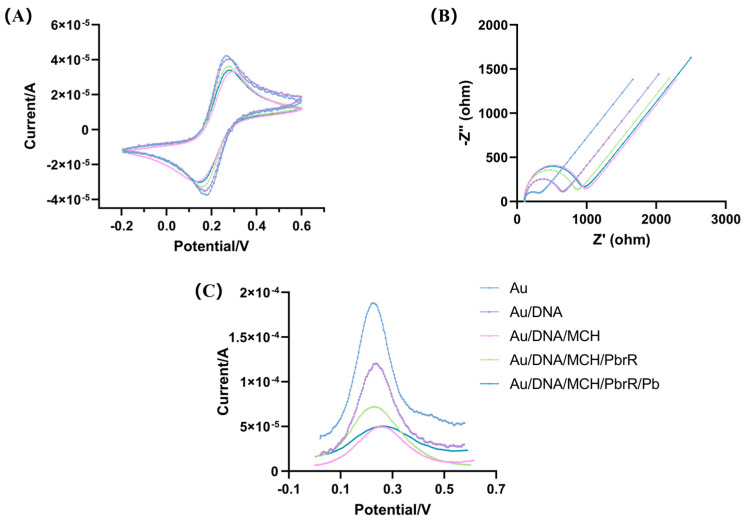
Characterization of the electrochemical biosensor for Pb^2+^ detection based on aTFs by (**A**) Cyclic voltammograms, (**B**) Nyquist diagrams and (**C**) Square-wave voltammogram.

CV and EIS are two wildly used characterization techniques in the development of electrochemical biosensors, utilizing potassium ferricyanide as a probe enables the detection of successful biomolecule modifications on the electrode surface [37,38]. CV is employed to characterize the modified electrode. As illustrated in Figure 2A, the smooth and polished surface of the gold electrode facilitates the efficient electron transfer of [Fe(CN)_6_]^3−/4−^, resulting in well-defined, symmetrical redox peaks. These peaks exhibit a high current response, which indicates the reversibility of the electrode reaction and the fast electron transfer rate. When DNA is modified on the gold electrode, the electron transfer rate of [Fe(CN)_6_]^3−/4−^ is impeded by the DNA strands’ negative charge, leading to a reduction in the redox peak current. As the non-specific binding sites on the electrodes are blocked by MCH, this also impedes the electron transfer rate of potassium ferricyanide, manifesting as a further decrease in the peak redox current. When PbrR was modified on the electrode, an increase in the redox peak current was observed, aligning with signal changes reported in the literature [39]. The presence of Pb^2+^ led to the detachment of PbrR from the DNA strand, resulting in a decrease in signal value. This decrease is attributed to the restoration of the DNA structure to its original state, which inhibits the electron transfer of [Fe(CN)_6_]^3−/4−^. 

EIS was employed to characterize the modified electrode. As depicted in Figure 2B, the polished gold electrode’s smooth surface facilitates the electron transfer of [Fe(CN)_6_]^3−/4−^, evident from a smaller semicircle in the high-frequency range and a reduced impedance value (Rct = 184 Ω). Modification of the electrode with DNA nucleic acid strands, which possess a negative charge, impedes this electron transfer, as indicated by an increased semicircle diameter and impedance (Rct = 488.3 Ω). Blocking non-specific binding sites on the electrode with MCH led to further increases in both the semicircle diameter and impedance (Rct = 804.3 Ω). Conversely, when PbrR was bound to the DNA strand, both the semicircle diameter and impedance decreased (Rct = 690.9 Ω), aligning with signal trends reported in the literature [39]. The presence of Pb^2+^ caused increases in both the semicircle diameter and impedance (Rct = 771.4 Ω), as Pb^2+^ binding to PbrR led to the detachment of PbrR from the DNA and a reversion to the DNA’s initial state. These EIS results correlated well with those from CV analyses.

Additionally, AFM was utilized to examine the assembly on the electrode surface (Figure S3). Following the immobilization of DNA and PbrR protein, the average surface roughness increased from 1.7 nm to 4.1 nm, paralleling the findings from previous studies [35]. This increase in roughness confirms the successful immobilization of the biorecognition elements. Collectively, these electrochemical and AFM analyses confirm the effective fabrication and assembly of the proposed biosensor.

Signal changes during electrode modification were monitored using SWV. As depicted in Figure 2C, the SWV signal from the bare gold electrode registered at 187 µA, Following DNA modification on the electrode, this signal decreased to 119 µA. The SWV signal was reduced to 49.5 µA. After modifying the electrode with PbrR, the SWV signal increased to 71.4 µA. Upon the introduction of 10 nM Pb^2+^, the signal decreased again to 50.3 µA, reverting to its initial state. We quantified the SWV signal change before and after Pb^2+^ detection using the equation ΔI = I_0_ − I, where I_0_ is the SWV signal post-PbrR modification, and I represents the signal change upon Pb^2+^ detection.

### 3.2. Optimization of the Electrochemical Biosensors

The performance of the biosensor is significantly influenced by the experimental conditions employed. To optimize biosensor performance, we investigated the impact of several critical factors, including the concentration of the DNA incubated on the electrode, the concentrations of PbrR, and the incubation time for Pb^2+^. These investigations are crucial for enhancing the sensor’s analytical performance and reliability.

**Figure 3:**
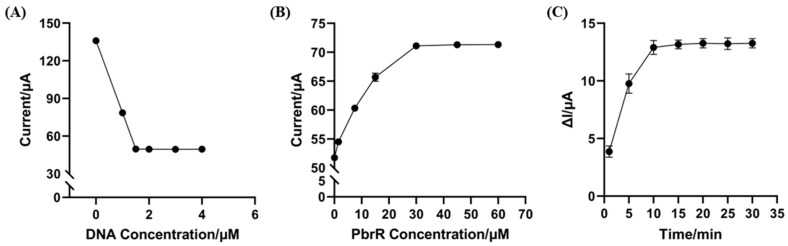
Optimization of electrochemical biosensors based on aTFs. (**A**) Signal response at different DNA concentrations. (**B**) Electrochemical signals of reactions with different PbrR concentrations. (**C**) Signal changes for different incubation times.

Firstly, the quantity of DNA on the electrode, which determines the extent of PbrR binding, directly impacts the quantitative detection of Pb^2+^. As shown in Figure S4, when the electrode was blocked with MCH, the trend of the SWV signal changes remained the same as before the MCH block. This indicates that the blocking of the nonspecific binding site with MCH only changed the value of the SWV signal. We incubated electrodes with varying DNA concentrations and then blocked non-specific binding sites with MCH to identify the optimal DNA concentration that yielded the most informative SWV signals. As illustrated in Figure 3A, the SWV signal values decreased as DNA concentration increased from 0 to 1.5 µM, suggesting that saturation was not achieved. However, from 1.5 to 4 µM, the SWV signals plateaued, indicating saturation at 1.5 µM. Therefore, the DNA concentration applied to the electrode was set at 1.5 µM.

Secondly, PbrR binds to DNA as dimers, which affects electron transfer at the electrode [36]. The incubation concentration of PbrR is a critical parameter that influences the biosensor’s performance, particularly when the DNA quantity on the electrode is fixed. Excessive PbrR can lead to waste and higher testing costs, while insufficient PbrR may compromise Pb^2+^ recognition and reduce the sensor’s analytical sensitivity. When the DNA concentration was fixed at 1.5 µM, the increasing PbrR concentrations led to rising SWV signals, as shown in Figure 3B. The SWV signal amplitude reached a plateau at a PbrR concentration of 30 µM, beyond which no significant changes were observed even as the concentration increased to 45 and 60 µM. Thus, the optimal incubation concentration of PbrR was determined to be 30 µM.

Lastly, the incubation time for Pb^2+^ was optimized to enhance the sensor’s sensitivity. We evaluated signal variations across different incubation times under consistent experimental conditions. As demonstrated in Figure 3C, the signal change increased progressively from 1 min to 10 min. Beyond 10 min, the signal change stabilized, showing no significant alterations for up to 30 min, which suggests that the maximum signal was achieved at an incubation time of 10 min. Therefore, the optimal incubation period for Pb^2+^ detection is 10 min. 

### 3.3. Sensitivity and Selectivity of the Electrochemical Biosensors 

**Figure 4:**
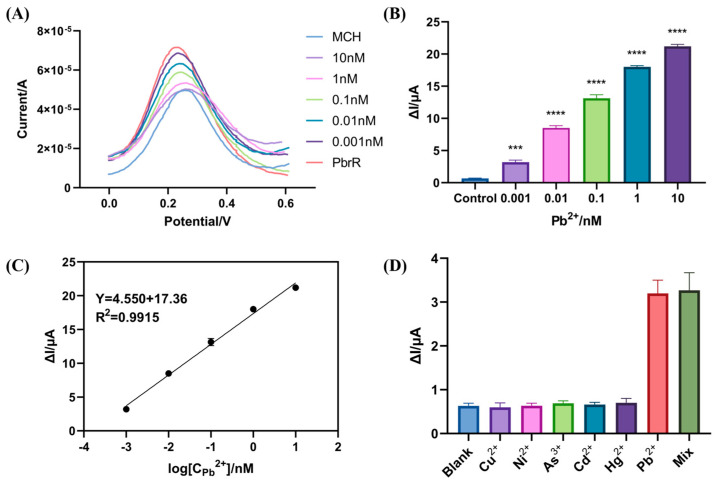
Quantitative analysis and selectivity of biosensors. (**A**,**B**). Signal change values of the biosensor after 10 min of incubation with different concentrations of Pb^2+^. (**C**). The linear relationship between the signal change and the concentration of Pb^2+^. (**D**). The biosensor signal changes after 10 min of incubation with different heavy metal ions. The concentration of all interfering heavy metal ions was 1 nM, and that of Pb^2+^ was 1 pM. Error bars; SD, *n* = 3. (* *p* < 0.05, ** *p* < 0.01, *** *p* < 0.001, and **** *p* < 0.0001).

To evaluate the detection performance of the prepared biosensors, 10 µL of the different concentrations of Pb^2+^ (0, 1 pM, 10 pM, 100 pM, 1 nM, 10 nM) were applied dropwise on the prefabricated electrochemical biosensors under optimal conditions. The electrochemical signals, detected using SWV, were utilized to determine the biosensors’ detection limits. As can be seen in Figure 4A, the distinct concentrations of Pb^2+^ produce different SWV signals, confirming a direct correlation between the signal change and Pb^2+^ concentration. This result implies that an elevation in the Pb^2+^ concentration led to the dissociation of PbrR from the DNA strand. The difference between the SWV signal value produced by PbrR and the SWV signal value after the addition of Pb^2+^ is obtained in Figure 4B, it can be seen that the SWV signal difference ΔI increases with the increase in Pb^2+^ concentration; this is indicative of a concentration-dependent response. The SWV signal changes caused by Pb^2+^ concentration (1 pM–10 nM) are significantly different from those of the control group, establishing that the limit of detection (LOD) of this biosensor is 1 pM; this confirms that the LOD of our biosensors match the requirement for water quality monitoring. Furthermore, Figure 4C shows that an excellent linear relationship exists between the SWV signal difference ΔI and the logarithm of the concentration of Pb^2+^ in the range of 1 pM to 10 nM, and the linear regression equation is Y = 4.550X + 17.36 (R^2^ = 0.9915), where Y is the SWV signal difference ΔI and X is the logarithmic value of Pb^2+^ concentration. 

Table 1 presents a comparison of various biosensors utilized for the detection of Pb^2+^, showcasing a range of recognition elements and their performance metrics. Among these, the electrochemical biosensor developed in this study, utilizing aTFs, stands out for its rapid detection capabilities and exceptional sensitivity. Building on our previous research detailed in Table 1, unlike the fluorescent biosensors previously studied, the electrochemical biosensor we established focuses more on ultra-sensitive detection. It is able to rapidly detect lead ions at low concentrations [27]. While the previous model detected Pb^2+^ at concentrations as low as 0.1 nM in 60 min, our current electrochemical approach achieves a lower detection limit of 1 pM in 10 min and simplifies the operational procedure by requiring only one electrochemical workstation without auxiliary temperature control and fluorescence excitation devices. The aTFs-based electrochemical biosensor take only 10 min to complete Pb^2+^ detection, which greatly reduces the time compared with other ultrasensitive detection methods [40,41]. Compared with the previous studies, our biosensor demonstrated an excellent LOD, despite the fact that some aptamer sensors had simpler surface modifications than our biosensor [42,43,44]. In comparison to other biosensors, our biosensor offers improvements in both cost-effectiveness and practical applicability for environmental monitoring, particularly highlighted by its superior sensitivity and streamlined usage. This comparative analysis underscores the biosensor’s exceptional capabilities, positioning it as a highly promising tool for the quantitative detection of Pb^2+^ in water quality monitoring applications.

In addition to high sensitivity, selectivity is a crucial criterion for Pb^2+^ detection in water. To investigate the selectivity of the aTFs-based electrochemical biosensors, five common heavy metal ions in polluted water, Cu^2+^, Ni^2+^, As^3+^, Cd^2+^, and Hg^2+^, were chosen as the potential interfering metal ions. The SWV signal difference generated by 1 pM Pb^2+^ was compared with those produced by the other metal ions at a concentration of 1 nM. As shown in the Figure 4D, the SWV signal difference ΔI of the target Pb^2+^ is higher at 1 pM compared to other metal ions. These results indicate that the electrochemical biosensor has a high selectivity for Pb^2+^. Given the potential existence of various heavy metal ions with Pb^2+^ in environmental water, it is necessary to verify that the detection electrochemical signal is accurate and not affected by other metals. Consequently, Pb^2+^ were mixed with other heavy metal ions for detection. The results confirm that the biosensor maintains its analytical accuracy even in the presence of multiple heavy metal ions. This exceptional selectivity is attributed to the specific recognition mechanism of Pb^2+^ by the PbrR [45]. Additionally, due to the properties of aTFs, there are other heavy metal ions and small molecules that are recognized by specific aTFs, such as MerR for mercury ions, TetR for tetracycline, NalC for pentachlorophenol [26,46,47].

### 3.4. Regeneration and Stability of the Electrochemical Biosensors

**Figure 5:**
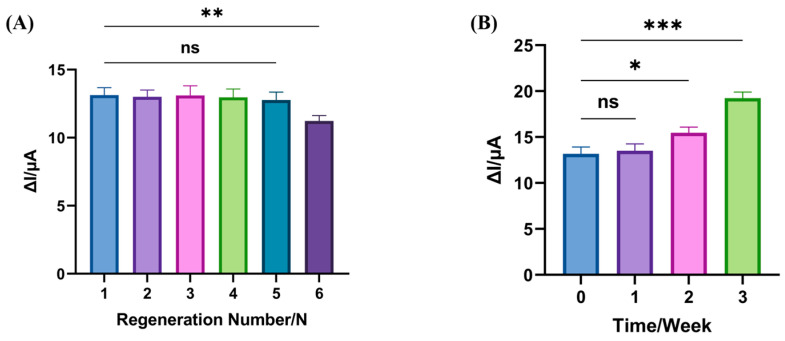
Regeneration and stability of biosensors. (**A**) Regeneration of biosensors. Where N represents the number of regenerations. (**B**) Stability of biosensors. Error bars; SD, *n* = 3. (* *p* < 0.05, ** *p* < 0.01, *** *p* < 0.001).

The regeneration capability of a biosensor is a critical factor for its practical application, underscoring its reusability in detection processes. To evaluate the regeneration performance of the constructed electrochemical biosensor, 10 µL of 0.5% SDS was applied dropwise onto the electrode surface and incubated for 30 min to remove the aTFs specifically bound to DNA, thereby initializing the electrochemical signal. This step is crucial for the regeneration of the biosensor. Subsequently, the biosensor was effectively reconstructed for subsequent detection by incubating the PbrR dropwise onto the electrode for 1 h to recombine with the DNA strand according to the properties of aTFs. As shown in Figure 5A, the SWV signal difference of the biosensor remained consistent after five consecutive regeneration cycles, indicating no significant discrepancy from the initial SWV signal change. However, a decrease in the SWV signal change was observed by the sixth cycle, indicating the beginning of a significant discrepancy from the initial signal change. This may occur because the modified DNA on the electrode surface becomes partially compromised after multiple regenerations, affecting the binding site’s recognition by PbrR. These findings demonstrate that our biosensor can be reliably reused up to five times for Pb^2+^ detection. Although each analysis requires only 10 min, it is imperative to note that the total duration for five successive analyses significantly exceeds a mere cumulative addition of individual analysis times due to the requisite 1.5 h sensor regeneration step (Table 1). The regeneration process, although it prolongs the total time of continuous detection, offers a significant reduction in the long-term operational costs by enabling the reusability of a single sensor, thereby decreasing the need for frequent replacements. This cost-saving is primarily reflected in the reuse of sensors, mitigating the expenses associated with the acquisition of new sensors. The cost per test was $0.57, including $0.35 for incubation PbrR and $0.22 for other reagent supplies, thereby enhancing the cost-effectiveness and sustainability of Pb^2+^ detection in environmental monitoring applications. 

In terms of stability, the modified Au/DNA/MCH/PbrR was stored at 4 °C and subsequently utilized for Pb^2+^ detection. As shown in Figure 5B, the signal change observed during the first week of detection was nearly identical to that of a freshly modified electrode, demonstrating consistent performance over this period. However, the SWV signal observed during the second week differed significantly from the initial measurements, indicating a decline in biosensor performance likely linked to the degradation of PbrR. These results confirm that the modified electrode maintains its effectiveness for detecting Pb^2+^ when stored at 4 °C for up to one week, thereby exhibiting commendable stability of biosensors.

### 3.5. Application of Biosensor for Pb^2+^ Detection in Actual Water Samples

We opted to detect natural samples of the Haihe River water to demonstrate the application of the aTFs-based electrochemical biosensors. Considering the low content of Pb^2+^ in the Haihe River water, spiking recovery experiments were conducted on real samples. As shown in Table 2, when the recoveries of Pb^2+^ spiked at spiked concentrations of 5, 7, and 10 nM were 93.3–106.6%, with the relative standard deviations (RSDs) within 2.25%, which demonstrated that the biosensors have the potential for application in detecting Pb^2+^ in complex environmental water samples with high accuracy. Meanwhile, the findings of the aTFs-based electrochemical biosensors were comparable with those of AAS, suggesting that this approach had excellent practical applicability in detecting actual water samples. 

## 4. Conclusions

In this study, by combining the electrochemical sensors with aTFs, we successfully developed an electrochemical biosensor based on aTFs for the detection of Pb^2+^. This represents the first integration of aTFs with electrochemical techniques for heavy metal detection. The aTFs-based electrochemical biosensor has a rapid recognition of and response to Pb^2+^, achieving quantitative detection within 10 min with a detection limit as low as 1 pM. The biosensor can be regenerated up to five times, ensuring the effective detection of Pb^2+^. Moreover, it maintains its efficacy for Pb^2+^ detection even after a week of storage at 4 °C. Excellent regeneration performance and stability can effectively reduce the cost of environmental monitoring, attributes that significantly reduce the testing cost in environmental monitoring. In the actual water samples, the recovery percentages were in the range of 93.3–106.6%. In conclusion, this study provides a cost-effective approach for Pb^2+^ detection, with the biosensor’s high sensitivity, specificity, reproducibility, and stability proving essential for environmental monitoring and public health protection. Furthermore, the successful development of this biosensor not only broadens the application of PbrR for the detection of Pb^2+^ but also presents a novel viewpoint for integrating other aTFs with electrochemical biosensors for specific target detection, including heavy metals and environmental contaminants. 

## Figures and Tables

**Table 1:** Comparison of the proposed biosensor with other reported works for Pb^2+^ detection.

Analytical Method	Recognition Element	Linear Range	Detection Limit	Detection Time	Reference	Notes
Electrochemical	aTFs	1 pM–10 nM	1 pM	10 min	This work	Sensor regeneration time 1.5 h
Fluorescence	aTFs	1–250 nM	0.1 nM	60 min	[27]	/
Electrochemical	aptamer	0.1–1000 nM	89.31 pM	30 min	[42]	
Electrochemical	aptamer	0.5 nM–5 µM	0.14 nM	15 min	[43]	
Electrochemical	G-quadruplex	0.01–200 nM	4.2 pM	60 min	[40]	
Electrochemical	DNAzymes	0.5 nM–5 µM	0.25 nM	25 min	[44]	
Electrochemical	DNA walker	0.05–1000 nM	4.65 pM	1.5 h	[41]	

**Table 2:** Spiked recovery detection of real samples by aTFs-based electrochemical biosensors.

Samples	Spiked (nM)	Detected (nM)	AAS (nM)	R.S.D. (%)
River water	5	5.33 ± 0.11	5.87 ± 0.15	2.21%
	7	7.0 ± 0.10	6.81 ± 0.02	1.51%
	10	10.24 ± 0.22	9.90 ± 0.03	2.25%

## Data Availability

Data are contained within the article.

## Supplementary Materials

The following supporting information can be downloaded at: https://www.mdpi.com/article/10.3390/bios14090446/s1, Table S1: PbrR Sequences; Table S2: DNA sequences; Text S1 and Figure S1: Characterization of gold electrode activation; Text S2 and Figure S2: EMSA verifies the binding of PbrR with DNA. Text S3 and Figure S3: AFM characterizes the assembly of biosensors. Figure S4: SWV signal changes with MCH blocking before and after.
