# Supplementary material for: Ultrasensitive Electrochemical Biosensors Based on Allosteric Transcription Factors (aTFs) for Pb2+ Detection
Source: Biosensors (Basel). 2024 Sep 18;14(9):446. doi: 10.3390/bios14090446 (PMC11430688; doi:10.3390/bios14090446)
Supplement: Supplementary file 1 [file biosensors-14-00446-s001.zip › biosensors-3151578-supplementary.pdf]

Ultrasensitive Electrochemical Biosensors Based on Allosteric Transcription Factors (aTFs) for Pb^{2+} Detection

Ningkang Yu [1,2,†], Chen Zhao [2,†], Xiaodan Kang [1,2], Cheng Zhang [2], Xi Zhang [2], Chenyu Li [2], Shang Wang [2], Bin Xue [2], Xiaobo Yang [2], Chao Li [2], Zhigang Qiu [2], Jingfeng Wang [2,*] and Zhiqiang Shen [1,2,*]

[1] College of Food Science and Technology, Shanghai Ocean University, Shanghai 201306, China; ynk0122@126.com (N.K.Y.); 13849918731@163.com (X.D.K.)

[2] Military Medical Sciences Academy, Tianjin 300050, China; zhaochen212@126.com (C.Z.); 15062724512@163.com (C.Z.); zhangxi0820@126.com (X.Z.); nk_lcy710430@hotmail.com (C.Y.L.); wsh847@163.com (S.W.); xue_bin04@163.com (B.X.); 18072712080@163.com (X.B.Y.); lc6628@163.com (C.L.); zhigangqiu99@gmail.com (Z.G.Q.);

* Correspondence: jingfengwang@hotmail.com (J.W.); tianjinszq922@sohu.com (Z.S.)

† These authors contributed equally to this work.

Table S1. PbrR Sequences[1]:

Name	Sequences
PbrR	MNIQIGELAKRTACPVVTIRFYEQEGLLPPPGRSRGNFRLYGE EHVERLQFIRHCRSLDMPLSDVRTLLSYRKRPDQDCGEVNM LLDEHIRQVESRIGALLELKHHLVELREACSGARPAQSCGILQ GLSDCVCDTRGTTAHPSD

Table S2. DNA sequences[1]:

Name	Modification	Sequences 5' - 3'
SH-DNA	5' thiol	TTTTTGTCTTGACTCTATAGTAACTAGAGGGTGTTAAATCGGCAA
cDNA	No	AAAAACAGAACTGAGATATCATTGATCTCCCACAATTTAGCCGTT
Bio-DNA	5' biotion	TTTTTGTCTTGACTCTATAGTAACTAGAGGGTGTTAAATCGGCAA
cDNA	No	AAAAACAGAACTGAGATATCATTGATCTCCCACAATTTAGCCGTT

Text S1 Electrode pretreatment

For the pretreatment of the electrodes, the gold electrodes (3 mm in diameter) were meticulously polished using a sequential gradient of alumina

powders: 1.0 μm, 0.3 μm, and 0.05 μm. This process was continued until the surface of the electrodes was uniformly smooth. Subsequently, the electrodes underwent ultrasonic cleaning in both ethanol and ultrapure water for 5 minutes each to remove any residual alumina powder. Electrode activation of the above electrodes was performed through cyclic voltammetry in 0.5 M H_2SO_4 at -0.2 ~ 1.6 V with a scan rate of 0.5 V/s, for a total of 10 cycles, until a steady-state redox curve was obtained. Cyclic voltammetric curves of electrode activation in **Figure S1**. Finally, the prepared electrode was rinsed with ultrapure water and dried with nitrogen.

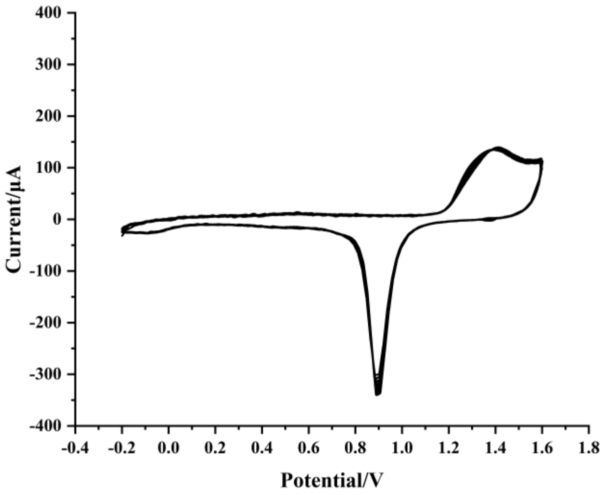

Figure S1. Characterization of gold electrode activation.

Text S2. Electrophoretic mobility shift assay (EMSA).

Following the approach of the article and with minor modifications[2]. Single stranded oligos containing PbrR binding sites (Bio-DNA) or control sequences (cDNA) were ordered from Sangon. Forward strands with 5′biotin were annealed to reverse strands by mixing to a final concentration of 10 μM in TE buffer (pH=6.8). heating to 95°C, then slowly letting them cool to room temperature.

To assess the binding of aTFs to DNA, we fixed DNA at 1 μM in reaction mixtures and added PbrR in graded concentrations to achieve various

PbrR/DNA ratios (ranging from 0 to 40). Reactions comprising various ratios of PbrR/DNA, water, transcription buffer, and poly (dI-dC) were incubated at 37 for 15 min. This step entailed the binding of aTFs with DNA. The binding of PbrR and DNA were observed using a 6.5% non-denaturing gel at 4 ◦C and 100 V in TBE (0.5×) buffer. The band signals were transferred for 1 h at 380 mV in TBE (0.5×) buffer using a Bio-Rad Power Pac HV (Bio-Rad, USA) to a positively charged nylon membrane (Millipore, USA). The band shifts were then detected and studied using chemiluminescence by employing a ChemiDoc XRS+ Gel Imaging System (Bio-Rad, USA).

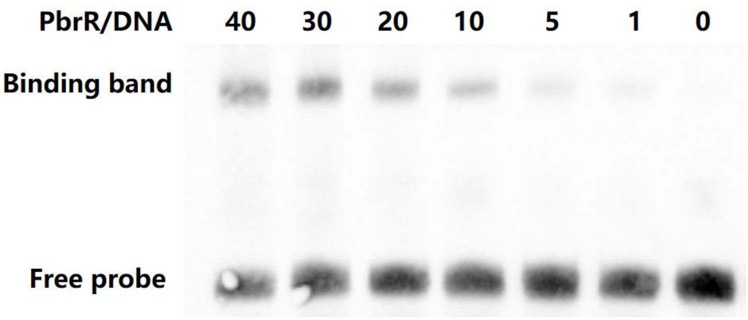

Figure S2. EMSA verifies the binding of PbrR with DNA.

Text S3. Atomic force microscopy (AFM) characterises the assembly of biosensors.

Atomic force microscopy (AFM) was performed with a Park FX40 (Park System, Korea) in Tapping mode of contact. The scanning range was 5*5 μm. Each sample was imaged in three separate sections. The average surface roughness was observed to escalate from 1.7 nm to 4.1 nm subsequent to the immobilization of DNA and the PbrR protein.

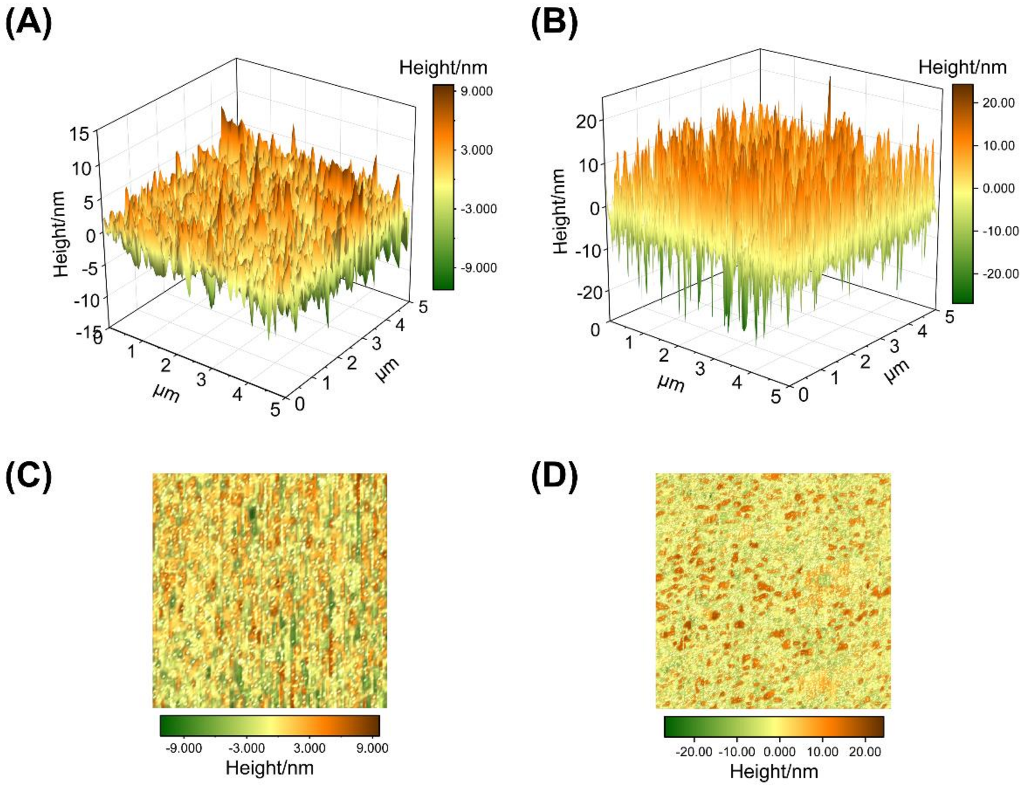

Figure S3. AFM characterises the assembly of biosensors (A) 3D AFM images of DNA modified electrodes. (B) 3D AFM images of DNA-PbrR modified electrodes. (C) 2D AFM images of DNA modified electrodes. (D) 2D AFM images of DNA-PbrR modified electrodes.

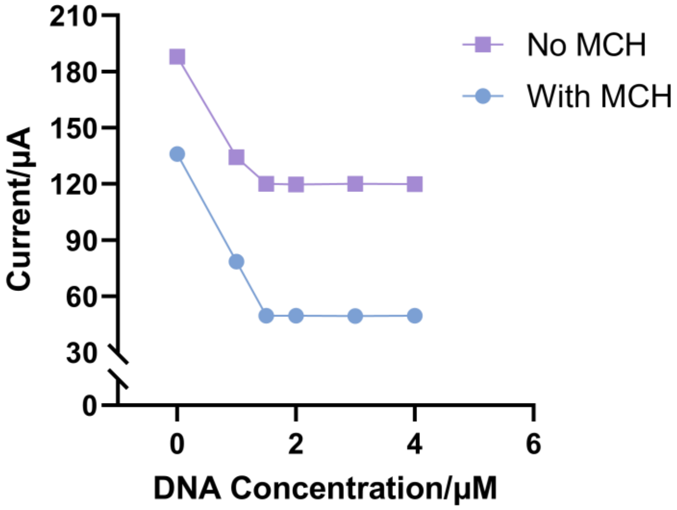

Figure S4. SWV signal changes with MCH blocking before and after.

Reference

1. Brown, N.L.; Stoyanov, J.V.; Kidd, S.P.; Hobman, J.L. The MerR family of transcriptional regulators. *FEMS Microbiol. Rev.* **2003**, *27*, 145–163. https://doi.org/10.1016/S0168-6445(03)00051-2.
2. Zhang, Y.; Zhao, C.; Bi, H.; Zhang, X.; Xue, B.; Li, C.; Wang, S.; Yang, X.; Qiu, Z.; Wang, J.; et al. A cell-free paper-based biosensor dependent on allosteric transcription factors (aTFs) for on-site detection of harmful metals Hg^{2+} and Pb^{2+} in water. *J. Hazard. Mater.* **2022**, *438*, 129499. https://doi.org/10.1016/j.jhazmat.2022.129499.
